# Obesidade Visceral e Hipertensão Sistólica como Substratos da Disfunção Endotelial em Adolescentes Obesos

**DOI:** 10.36660/abc.20190541

**Published:** 2021-04-08

**Authors:** Maria Fernanda Hussid, Felipe Xerez Cepeda, Camila P. Jordão, Rafaela R. P. Lopes-Vicente, Leslie Virmondes, Keyla Y. Katayama, Ezequiel F. de Oliveira, Luis V. F. Oliveira, Fernanda Marciano Consolim-Colombo, Ivani Credidio Trombetta

**Affiliations:** 1 Universidade Nove de Julho São PauloSP Brasil Universidade Nove de Julho (UNINOVE), São Paulo, SP - Brasil.; 2 Universidade de São Paulo Faculdade de Medicina Hospital das Clínicas São PauloSP Brasil Instituto do Coração (InCor), Hospital das Clínicas da Faculdade de Medicina da Universidade de São Paulo, São Paulo, SP - Brasil.; 3 Centro Universitário de Anápolis AnápolisGO Brasil Centro Universitário de Anápolis (UniEvangélica), Anápolis, GO - Brasil.

**Keywords:** Adolescente, Obesidade, Síndrome Metabólica, Hipertensão, Diabetes, Circunferência da Cintura, Síndrome da Apneia Obstrutiva do Sono, Endotélio, Fatores de Risco

## Abstract

**Fundamento:**

A obesidade afeta a adolescência, podendo levar à síndrome metabólica (SM) e disfunção endotelial, um marcador precoce de risco cardiovascular. Apesar de a obesidade ser fortemente associada à síndrome da apneia obstrutiva do sono (SAOS), ainda não está claro o papel da SAOS na função endotelial em adolescentes obesos.

**Objetivo:**

Investigar se a obesidade durante a adolescência leva à SM e/ou SAOS e causa disfunção endotelial nesses indivíduos. Além disso, estudamos a possível associação dos fatores de risco para SM e do índice de apneia e hipopneia (IAH) com disfunção endotelial.

**Métodos:**

Estudamos 20 adolescentes obesos sedentários (AO; 14,2±1,6 anos, 100,9±20,3kg), e 10 adolescentes eutróficos (AE, 15,2±1,2 anos, 54,4±5,3kg) pareados por sexo. Avaliamos os fatores de risco para SM (critérios da Federação Internacional de Diabetes), função vascular (dilatação mediada pelo fluxo, DMF), capacidade funcional (VO^2^pico) e presença de SAOS (IAH > 1 evento/hora, pela polissonografia). Consideramos um p<0,05 como estatisticamente significativo.

**Resultados:**

AO apresentaram maior circunferência da cintura (CC), gordura corporal, triglicerídeos, pressão arterial sistólica (PAS) e diastólica (PAD), maiores níveis de LDL e menores HDL e VO^2^pico em comparação a AE. Não houve diferença no IAH entre os grupos. AO apresentaram menor DMF que AE (6,17±2,72 vs. 9,37±2,20%, p=0,005). Observou-se uma associação entre DMF e CC (R=-0,506, p=0,008) e entre DMF e PAS (R=-0,493, p=0,006).

**Conclusão:**

Em adolescentes, a obesidade associou-se à SM e causou disfunção endotelial. CC e PAS aumentadas poderiam estar envolvidas nessa alteração. SAOS foi detectada na maioria dos adolescentes independentemente de obesidade. (Arq Bras Cardiol. 2021; 116(4):795-803)

## Introdução

A obesidade tem aumentado rapidamente em todo o mundo e é considerada um fator de risco para doenças crônicas não transmissíveis. Crianças e adolescentes têm sido seriamente impactados por essa tendência, particularmente em países em desenvolvimento, segundo a Organização Mundial de Saúde (OMS).^1^ Ao avaliar o estado nutricional de escolares com idade entre 13 e 17 anos por meio do índice de massa corporal (IMC), observa-se que 23,7% da população do sexo masculino encontra-se com sobrepeso, e 8,3% são obesos.^2^ A preocupação da elevada prevalência de obesidade em crianças e adolescentes baseia-se no fato dessa condição ser um possível preditor de obesidade na fase adulta, levando a um risco aumentado de doenças crônicas, tais como diabetes tipo 2, síndrome metabólica (SM) e doenças cardiovasculares (DCV).^3^

De fato, está claro na literatura que a obesidade está positivamente associada com a incidência de SM.^4^ Estudos realizados com adolescentes na puberdade mostraram uma prevalência de SM variando de 25 a 30%.^5^ Nesse estudo, os autores encontraram que a circunferência da cintura (CC) foi um preditor de SM, com um aumento de 11% no risco de SM a cada 1 cm de incremento na circunferência abdominal.^5^

Já está bem estabelecido que o marcador mais precoce de aterosclerose é disfunção endotelial,^6^ a qual pode ser encontrada tanto na hipertensão como na aterosclerose. A disfunção endotelial também está envolvida em processos fisiológicos e patológicos, incluindo inflamação, resistência insulínica e obesidade, entre outras doenças.^6^

A dilatação mediada pelo fluxo (DMF) na ultrassonografia é um método amplamente utilizado para avaliar função endotelial, o que pode ser um preditor de eventos cardiovasculares tanto em indivíduos assintomáticos como em indivíduos com DCV. Uma mudança na DMF pode ter valor prognóstico em humanos.^7^

As doenças respiratórias do sono estão entre as consequências da obesidade, incluindo a síndrome da apneia obstrutiva do sono (SAOS). A SAOS é o distúrbio respiratório do sono mais comum, com uma prevalência de 1 a 4% na infância, com pico na faixa etária de 2 a 8 anos.^8^ Em crianças obesas, essa porcentagem pode atingir 36%.^9^ A SAOS tem sido correlacionada com obesidade, promovendo um ambiente de inflamação moderada e crônica. Pacientes com SAOS apresentam episódios de hipóxia e despertares recorrentes durante o sono devido à atividade aumentada do sistema nervoso simpático.^9^ Trombetta et al.,^10^ encontraram que pacientes com SM e SAOS apresentavam maiores níveis de pressão arterial em comparação àqueles com SM e sem SAOS. Atividade simpática aumentada e disfunção do barorreflexo foram observadas nesses pacientes com SAOS associada à SM.^10^ A associação entre obesidade e SAOS poderia aumentar o risco de disfunção endotelial.^11^

No presente estudo, adolescentes obesos foram comparados com adolescentes eutróficos quanto à antropometria, composição corporal, parâmetros bioquímicos e reatividade vascular, e apneia do sono. Nosso objetivo foi investigar se a obesidade durante a adolescência: 1) leva à SM e/ou SAOS; e 2) causa disfunção endotelial. Além disso, estudamos a possível associação da SM ou o índice de apneia-hipopneia (IAH) com a disfunção endotelial.

## Métodos

### Comitê de Ética

O estudo foi aprovado pelo comitê de ética em pesquisa da Universidade Nove de Julho (UNINOVE) sob número 973.013, CAAE: 41899215.0.0000.5511. Os pais ou responsáveis dos adolescentes foram informados sobre os procedimentos do estudo e deram consentimento informado por escrito. Os adolescentes também foram informados sobre todos os procedimentos e deram consentimento por escrito.

### Sujeitos

Este foi um estudo transversal. Adolescentes com idade entre 12 e 17 anos atendidos no ambulatório da UNINOVE foram convidados para participarem no estudo de acordo com os critérios de inclusão e de exclusão. Incluímos no estudo adolescentes pós-púberes segundo classificação de Tanner (M4 para meninas ou menarca, e G4 para meninos),^12^^,^^13^ eutróficos ou obesos, sedentários, sem tratamento dietético ou medicamentoso para obesidade, com ou sem SM. Os critérios de exclusão foram adolescentes que não se encontravam no estágio pós-púbere, com sobrepeso, e aqueles com suspeita ou diagnóstico confirmado de alguma síndrome genética ou distúrbio neuroendócrino, tais como hipotiroidismo descontrolado e diabetes tipo 1. Pacientes com distúrbios alimentares (anorexia nervosa, bulimia nervosa, ou distúrbio alimentar não específico) também foram excluídos. Um total de 20 adolescentes obesos (AO) e 10 adolescentes eutróficos (AE) foram estudados.

O diagnóstico de SM foi estabelecido utilizando os critérios da Federação Internacional de Diabetes (IDF,*International Diabetes Federation*). Obesidade central foi definida como CC≥94 cm para homens e ≥80 cm para mulheres), combinada com dois destes quatro critérios diagnósticos: (1) lipoproteína de alta densidade (HDL) < 40mg/dL (<1,03 mmol/L) em homens e <50 mg/dL (1,29mmol/L) em mulheres; (2) glicemia de jejum ≥100mg/dL (≥5,6 mmol/L); (3) triglicerídeos de jejum (TG) ≥ 150 mg/dL (>1,69 mmol/L); e (4) pressão arterial sistólica (PAS) ≥130 e diastólica (PAD) ≥85 mmHg.^14^^,^^15^

### Medidas

#### Medidas Antropométricas e Composição Corporal

Foram avaliados peso e altura, e em seguida calculado o IMC. O IMC foi expresso em escore padrão (escore-z); peso normal foi definido como um z-score entre −2 e +1; sobrepeso como um z-score entre +1 e +2; e obesidade como >+2. A avaliação da composição corporal foi realizada por análise de bioimpedância elétrica (RJL, Quantum II model, Clinton Twp, Mi, EUA). A CC e a circunferência do pescoço (CP) foram medidas conforme descrito anteriormente.^16^^,^^17^

#### Pressão Arterial

PAS e PAD foram medidas utilizando-se manguito de tamanho apropriado.^18^^–^^20^

#### Análise Sérica

As amostras de sangue foram coletadas pela manhã após 12 horas de jejum. Foram determinadas concentrações de glicose, TG, colesterol total, HDL-colesterol, lipoproteína de baixa densidade (LDL-colesterol), razão TG/HDL-c e razão LDL-c/HDL-c.

### Polissonografia Noturna

Foi realizada polissonografia de noite inteira (monitoramento padrão – nível 1) utilizando-se um sistema de análise ambulatorial do sono (Embla Somnologica Studio - EMBLA A10, versão 3.1.2.; Flagahf Medical Devices, Iceland), conforme descrito anteriormente.^21^^,^^22^ Uma vez que havia adolescentes com idade de 12 anos no estudo, escolhemos os critérios de classificação da AASM (*American Academy of Sleep Medicine*) para SAOS em crianças.^23^ SAOS foi definida como um IAH>1 evento/hora; um IAH≥1-4,99 foi considerado SAOS leve; um IAH de 5 a 9,99 foi considerado SAOS moderado, e um IAH ≥10 SAOS grave.^9^

Em crianças, uma apneia é pontuada quando o pico do sinal cai ≥ 90% da linha de base pré-evento. A hipoventilação é pontuada quando o CO^2^ arterial (ou substituto) é> 50 mm Hg por> 25% do tempo total de sono. O IAH é a soma do número total de eventos respiratórios (apneias mais hipopneias) por hora de sono. O índice de despertar foi definido como o número médio de despertares por hora de sono. A dessaturação de oxigênio (nadir de SaO^2^) foi definida como a menor saturação de oxigênio da hemoglobina registrada por oximetria de pulso.^23^

### Teste de Esforço Cardiopulmonar (TECP)

O TECP foi realizado em uma esteira conectada a um sistema composto de um módulo de análise de gás, acoplado a um analisador de onda/módulo de fluxo, com medida a cada respiração (*breath-by-breath mode*) (BreezeCardiO2 System microcomputer; Medical Graphics Corporation-MGC, St. Paul, Mo, EUA), com protocolo de rampa. O TEPC permite medir a capacidade funcional (VO^2^ pico) como o VO^2^ máximo alcançado ao final do teste.^24^^,^^25^

### Hiperemia Reativa

#### Dilatação Mediada pelo Fluxo (DMF)

A DMF foi realizada por ultrassom vascular de alta resolução (Vivid i, GE Medical Systems, Tirat Carmel, Israel), medindo-se a dilatação do vaso (dilatação dependente do endotélio) da artéria braquial, como descrito anteriormente.^26^ Em resumo, os participantes deitaram-se em repouso por pelo menos 10 minutos, e o primeiro registro em repouso foi realizado. Em seguida, um fluxo aumentado foi induzido utilizando um esfigmomanômetro, com manguito colocado distalmente à artéria braquial no antebraço, insuflado até uma pressão superior à pressão sistólica (aproximadamente 20-30 mmHg) durante 5 minutos. O manguito foi desinflado, e foram registrados o fluxo e a dilatação do vaso, representada pela tensão de cisalhamento. A diferença entre o diâmetro basal e o diâmetro após a dilatação foi avaliada.

#### Índice de Hiperemia Reativa (IHR) por Tonometria Arterial Periférica

A função endotelial foi avaliada pela medida do IHR por tonometria arterial periférica (Endo-PAT2000; Itamar Medical, Cesareia, Israel) conforme descrito anteriormente.^27^ Esse método avalia a função endotelial microvascular.^28^

Para avaliação tanto da DMF como do IHR, os adolescentes foram orientados a fazerem jejum de 4 a 6 horas, e evitarem consumo de cafeína, chocolate, alimentos gordurosos, e praticarem exercício no dia do exame.

### Análise Estatística

A análise estatística foi realizada usando o programa SPSS20 Statistics (IBM Corp., Armonk, NY, EUA). O tamanho da amostra foi calculado pelo website http://www.openepi.com. Levamos em consideração um poder de 80%, com erro tipo 1 de 0,05 (bicaudal). Usamos variáveis da função endotelial (IHR e DMF) como desfecho primário. Nós escolhemos o maior número de indivíduos, 30 adolescentes para o estudo. A normalidade das amostras foi testada pelo teste de Kolmogorov-Smirnov. As variáveis paramétricas foram expressas em média ± desvio padrão (DP) e variáveis não paramétricas foram expressas em mediana e intervalo interquartil. Os dados categóricos foram descritos em valores absolutos e porcentagem da amostra total. As variáveis paramétricas dos grupos de AO e AE foram comparadas pelo teste t de Student independente e as variáveis não paramétricas foram comparadas pelo teste de Mann-Whitney. A variáveis categóricas foram analisadas pelo teste do qui-quadrado e a correlação de Pearson usada para analisar a correlação entre variáveis de fatores de risco tais como CC e pressão arterial, bem como porcentagem da DMF. Valores de probabilidade menores que p<0,05 foram considerados estatisticamente significativos.

## Resultados

**Figura 1:**
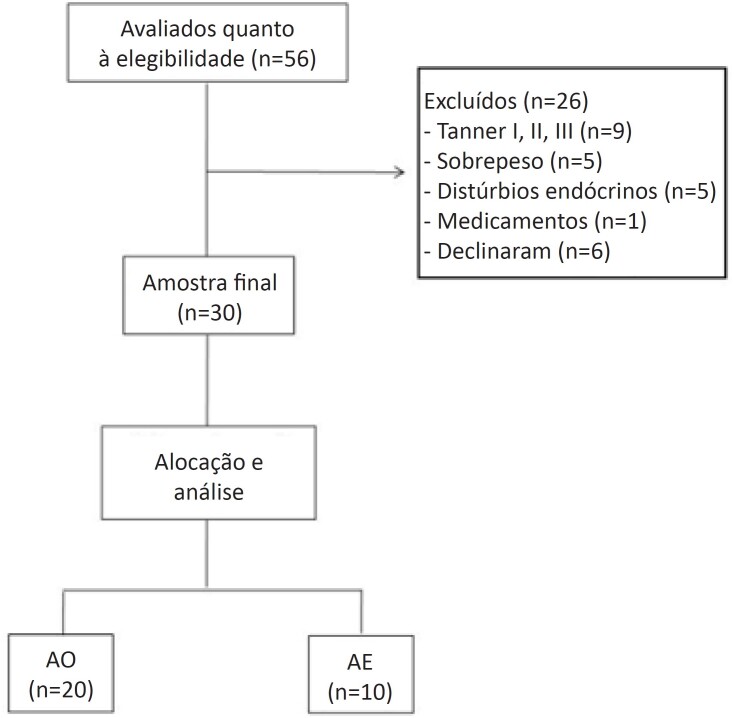
Fluxograma de inclusão dos participantes no estudo; AO: adolescentes obesos; AE: adolescentes eutróficos.

**Figura 2:**
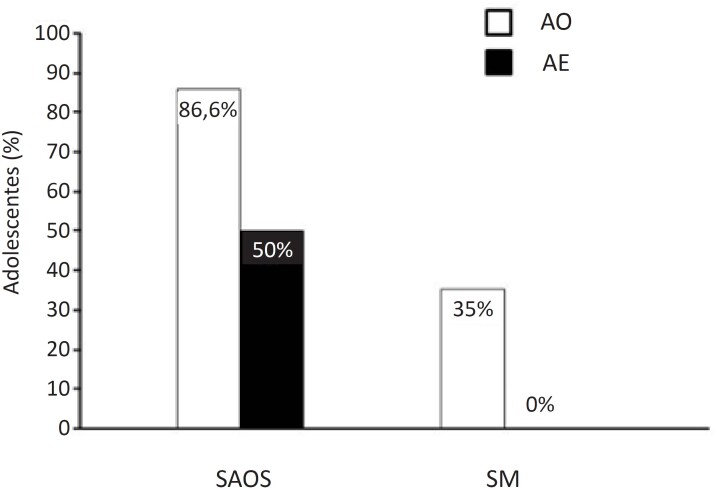
Porcentagem de síndrome da apneia obstrutiva do sono (SAOS) e síndrome metabólica (SM) em adolescentes obesos (AO) e adolescentes eutróficos (AE).

Inicialmente recrutamos 56 adolescentes, 26 deles foram excluídos – nove eram classificados como escala de Tanner I, II ou III; cinco estavam em sobrepeso; cinco apresentavam distúrbios endócrinos; um usava medicamento e seis recusaram a participar do estudo. Nossa amostra final foi composta de 30 adolescentes. Assim, estudamos 20 AO (10 do sexo masculino) e 10 AE (5 do sexo masculino) (Figura 1). Sete adolescentes do grupo AO apresentavam SM (35%) e nenhum AE apresentava SM (Figura 2).

Na Tabela 1, descrevamos as medidas antropométricas e de composição corporal dos participantes. Ambos os grupos foram similares na distribuição de sexo e de idade. Como esperado, AO apresentavam maior peso corporal, IMC, CP e CC. Em relação à composição corporal, observou-se menor porcentagem de água corporal e massa magra e maior massa gorda no grupo de AO.

**Tabela 1:** Dados antropométricos e de composição corporal em adolescentes obesos e eutróficos

	AO (n=20)	AE (n=10)	p
Sexo (M/F)	10/10	5/5	1
Idade (anos)	14,2±1,6	15,2±1,2	0,075
Peso (kg)	100,1±20,3	54,4±5,3	<0,001
Altura (m)	1,67±0,08	1,65±0,6	0,760
IMC (kg/m²)	35,9±6,2	19,9±1,8	<0,001
CP (cm)	38,3±3,6	31,9±1,8	<0,001
CC (cm)	107,9 [100-114,5]	67,5 [66,4-73,7]	<0,001
Água corporal (%)	45,4±3,8	56,5±4,6	<0,001
Gordura corporal (%)	38±5,2	22,9±6,3	<0,001
Massa magra (%)	62±5,2	77,1±6,3	<0,001

Dados paramétricos apresentados em média ± desvio padrão. Dados não paramétricos apresentados em mediana e intervalo interquartil. AO: adolescentes obesos; AE: adolescentes eutróficos; IMC: índice de massa corporal; CP: circunferência do pescoço; CC: circunferência da cintura.

Dados sobre fatores de risco cardiovasculares nos grupos AO e AE são apresentados na Tabela 2. Não houve diferença nos níveis de HDL ou de glicemia entre os grupos. Em comparação aos AE, os AO apresentaram maior PAS e maior PAD, além de níveis maiores de TG. LDL-c, TG/HDL-c, colesterol não HDL, LDL/HDL-c, e colesterol total. No TECP, o grupo de AO apresentou menor VO^2^pico em comparação aos AE. Resultados da polissonografia revelaram que um O^2^ mínimo mais baixo AO em comparação a AE. Não houve diferença no índice de despertares ou no IAH entre os grupos (Tabela 2). No entanto, a maioria dos adolescentes do grupo AO (86,6%) e 50% dos AE apresentaram IAH ≥1 evento/h (Figura 2).

**Tabela 2:** Fatores de risco cardiovascular em adolescentes obesos e eutróficos

	OA (n=20)	EA (n=10)	p
PAS (mmHg)	120 [110-127,5]	110 [100-110]	0,001
PAD (mmHg)	75 [70-80]	65 [60-70]	0,005
Glicemia (mg/mL)	84,9±5,4	89,3 ±7,2	0,140
TG (mg/dL)	120,5±48,3	71,1±28,8	0,020
HDL-c (mg/dL)	41,2±7,7	48,4 ±10,7	0,079
TG/HDL-c ratio	3,1±1,6	1,6±1	0,011
LDL-c (mg/dL)	97,5±25,7	69,9±22,2	0,015
nHDL-c (mg/dL)	121,5±27,5	83,2±26,2	0,004
LDL/HDL-c radio	2,4±0,8	2,6±0,7	0,007
Colesterol total (mg/dL)	162,7±28,7	132,5±24,1	0,016
**Polissonografia de noite inteira**			
IAH (events/h)	5,6±3,8	3,1±3,4	0,121
O^2^ Sat mínimo (%)	90 [81-90]	92,5 [88,5-93]	0,026
Índice de despertares	50,6±18,1	50±9,3	0,943
**Teste de esforço cardiopulmonar**			
VO^2^pico (mL/kg/min)	30,6±7,7	23,4±5,9	0,022

Dados paramétricos apresentados em média ± DP. Dados não paramétricos expressos em mediana e intervalo interquartil. AO: adolescentes obesos; AE: adolescentes eutróficos; PAS: pressão arterial sistólica; PAD: pressão arterial diastólica; TG: triglicerídeos; HDL: lipoproteína de alta densidade; LDL: lipoproteína de baixa densidade.

**Figura 3:**
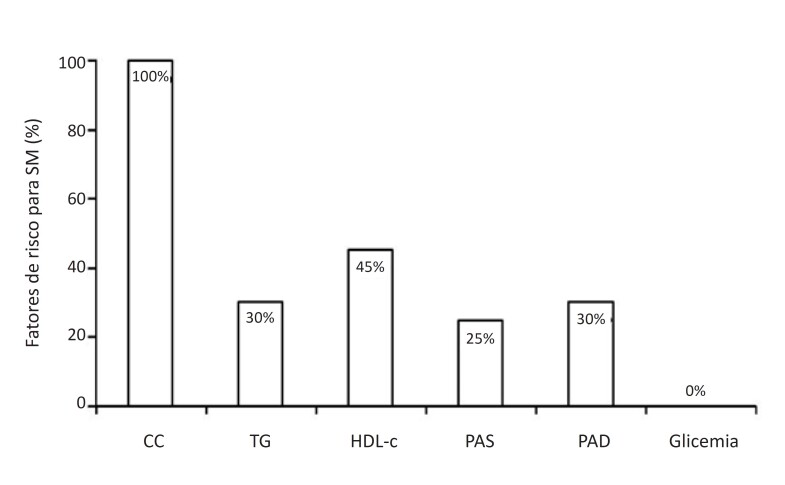
Porcentagem de fatores de risco para síndrome metabólica (SM) em adolescentes obesos; CC: circunferência da cintura; TG: triglicerídeos; HDL: lipoproteína de alta densidade; PAS: pressão arterial sistólica; PAD: pressão arterial diastólica.

**Figura 4:**
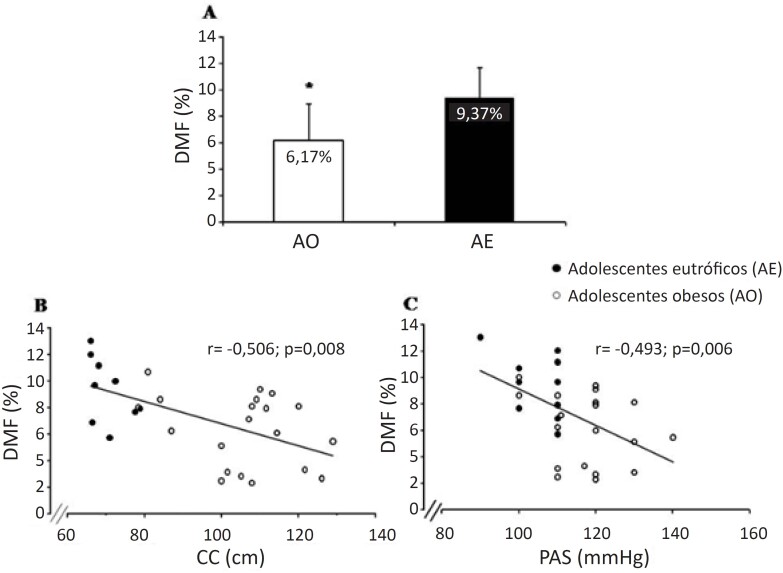
Hiperemia reativa por dilatação mediada pelo fluxo (DMF) (A); coeficiente de correlação entre DMF e circunferência da cintura (CC) (B); e coeficiente de correlação entre DMF e pressão arterial sistólica (PAS) em adolescentes obesos (AO) e adolescentes eutróficos (AE) (C). * p<0,005.

Na Figura 3, apresentamos a prevalência dos fatores de risco para SM segundo a IDF.^15^ Na Figura 4A, apresentamos as análises da DMF. Em um participante do grupo AO, foi detectada bifurcação da artéria braquial, e decidimos excluir esse dado das análises. As análises da DMF mostraram que os AO reatividade vascular mais baixa das grandes artérias em comparação ao grupo AE (6,17±2,72% vs. 9,37±2,20%, p=0,005). Diante disso, exploramos a associação entre DFM e os fatores de risco para SM, e encontramos uma associação entre CC e DMF (R=-0,506, p=0,008; Figura 4B), e entre PAS e DMF (R=-0,493, p=0,006; Figura C).

## Discussão

O principal achado do presente estudo é o fato de que AO apresentam disfunção endotelial, indicada por uma reatividade vascular reduzida. Além disso, com base nas análises de correlação, podemos sugerir que a CC e os níveis de PAS podem ser preditores desta disfunção.

A obesidade e a SM aumentam o risco de disfunção endotelial, e a SAOS contribui para esse agravamento. Contudo, apesar da alta prevalência de SAOS em AO, não houve diferenças na presença de SAOS e de IAH entre os grupos estudados. Podemos sugerir que, em adolescentes, outros fatores além da obesidade, tais como rinite alérgica, asma e hipertrofia adenotonsilar, possam contribuir com a SAOS. Esses achados foram encontrados em outros estudos.^29^ Assim, no presente estudo, a DMF deficiente não esteve associada à SAOS, e a presença de SAOS não pôde ser atribuída à obesidade ou à SM. Nós especulamos que a SM potencializou a disfunção endotelial no grupo de AO, uma vez que 35% dos pacientes obesos na nossa amostra apresentaram SM.

A aterosclerose e as manifestações clínicas da DCV originam-se na infância,^30^ e sua detecção precoce é muito importante para sua prevenção. A disfunção endotelial é considerada um sinal precoce de aterosclerose em crianças com fatores de risco para DCV, e pode ser revertida por intervenções que visam diminuir o risco cardiovascular.^30^

A DMF com hiperemia reativa é um método não invasivo que avalia a vasodilatação dependente de endotélio, mediada por óxido nítrico (NO), e é um método diagnóstico adequado para o grupo etário estudado. Uma meta-análise identificou que um aumento em 1% na DMF aumenta o risco futuro de eventos cardiovasculares em 13%.^3^ Há evidência de que crianças e adolescentes obesos apresentam menor complacência e distensibilidade vascular em comparação àqueles com peso normal.^3^ Tal fato poderia explicar os níveis mais elevados de pressão sanguínea nos AO.^31^

Um estudo conduzido com adultos relatou uma DMF de 9,4% ± 4,7%.^32^ Uma meta-análise de Dias et al.,^3^ identificou uma DMF de 6,0% ± 0,69% em adolescentes obesos, em comparação a 12,32% ± 3,14% em adolescentes eutróficos.^3^ Esse dado corrobora nossos achados, que mostraram uma reatividade vascular reduzida em AO em comparação a AE (6,17 ± 2,72% e 9,37 ± 2,20%, respectivamente).

A DMF é uma medida indireta da biodisponibilidade do NO,^26^^,^^28^^,^^32^ uma vez que ela estimula um meio isquêmico e, em seguida, vasodilatação. A oclusão dos vasos leva à liberação de adenosina, fator hiperpolarizante derivado do endotélio, íons de hidrogênio, entre outras substâncias, com o objetivo de restaurar a perfusão sanguínea via dilatação da microcirculação. Neste método, quando o manguito é esvaziado, a circulação é restaurada com aumento do aporte sanguíneo para a região isquêmica, causando “hiperemia reativa”. A tensão de cisalhamento, causada pelo aumento do fluxo sanguíneo, e sua velocidade, leva à liberação de substâncias vasodilatadoras pelo endotélio, tais como o NO, via ativação da enzima óxido nítrico sintase endotelial (eNOS), e consequente relaxamento da musculatura lisa vascular e aumento no diâmetro arterial. Uma capacidade de relaxamento mais baixa leva à disfunção endotelial.^26^^,^^28^^,^^32^ Uma menor vasodilatação pode ocorrer nos meninos em comparação às meninas, além de existir uma variação de função endotelial durante o ciclo menstrual.^28^ Portanto, nós realizamos os exames na primeira fase do período menstrual. O EndoPAT® foi avaliado por Radke et al. em relação aos estágios da puberdade.^33^ Um IHR mais baixo foi observado na puberdade em comparação aos estágios de Tanner 4 e 5, com variação entre 1,11 e 1,70. O ponto de corte para adultos é 1,35, o que poderia ser usado para identificar os indivíduos com disfunção endotelial na microcirculação. Essa técnica foi desenvolvida para ser independente de examinador. Sabe-se que, devido à localização do manguito a ser inflado, a vasodilatação da microcirculação obtida não é totalmente dependente de NO. Assim, enquanto o EndoPAT® mede a função endotelial da microcirculação, e DMF avalia a função endotelial das artérias condutoras. É possível que os resultados sejam comparativamente discrepantes, porém complementares, uma vez que os métodos avaliam sistemas diferentes.^28^ Nos adolescentes avaliados neste estudo, não houve diferença nesta medida entre AO e AE e, assim, não conseguimos identificar complementariedade entre EndoPAT® e DMF.

Outro fator relevante no estudo foi a correlação entre CC e reatividade vascular. Uma CC aumentada é um preditor de risco para DCV,^15^^,^^34^ conhecido como “síndrome da adiposidade visceral”.^4^^,^^34^ Com o aumento da adiposidade visceral, ocorre um aumento nos depósitos de gordura patogênica e piora na reatividade vascular. A distribuição da gordura visceral é um fator preditivo de hipertensão, maior que o aumento generalizado em gordura. O sistema nervoso simpático parece estar relacionado a diferentes componentes da síndrome da adiposidade a visceral, gerando um aumento real na atividade simpática,^34^ e um risco aumentado de hipertensão nesses pacientes.

Apesar de o IAH não ter sido diferente entre os dois grupos estudados, houve uma prevalência de 86,6% e 50% de SAOS e de 35% e 0% de SM nos grupos AO e AE, respectivamente. A maior presença de SAOS e SM pode ter contribuído para o aumento de PAS nesse grupo, o que pode ter sido modulado por um tônus simpático aumentado. Esse fato já foi observado por Trombetta et al., que relataram maior atividade e barorreflexo reduzido em pacientes adultos com SM associada à SAOS.^10^

No presente estudo, observamos que AO exibiram VO^2^pico reduzido, sugerindo um risco cardiovascular aumentado. De fato, existe forte evidência de que a obesidade associa-se com um pior prognóstico em adolescentes com capacidade funcional reduzida e presença de comorbidades cardiometabólicas. Medidas preventivas são necessárias nesses indivíduos com disfunção endotelial, estimulando a prática de atividade física e dieta saudável visando a redução da CC e da pressão arterial.

### Limitações

Nosso estudo tem várias limitações. Primeiro, considerando que, nas meninas, existe variação da função endotelial durante o ciclo menstrual^28^ e, apesar de termos realizado o teste na primeira fase do ciclo menstrual, algumas tiveram apenas a menarca e, portanto, não apresentavam regularidade ou conhecimento do ciclo. Segundo, uma vez que não existe consenso sobre os critérios diagnósticos para SAOS em indivíduos com idade entre 13 e 18 anos, no presente estudo, similar a outros,^35^^,^^36^ utilizamos valores pediátricos. Os critérios usados para a idade de até 13 anos foram estendidos para até 18 anos, com base no manual da AASM para pontuação de sono e eventos associados.^23^

## Conclusão

Na amostra estudada, a obesidade foi um importante fator de risco para o desenvolvimento de SM, e levou à disfunção endotelial, a qual é o ponto inicial da formação da placa de ateroma. Além disso, CC e PAS aumentadas são preditoras de disfunção endotelial em adolescentes. A SAOS estava presente na maioria dos adolescentes, independentemente da obesidade.
