# Supplementary material for: Obesidade Visceral e Hipertensão Sistólica como Substratos da Disfunção Endotelial em Adolescentes Obesos
Source: Arq Bras Cardiol. 2021 Apr 8;116(4):795–803. [Article in Portuguese] doi: 10.36660/abc.20190541 (PMC8121384; doi:10.36660/abc.20190541)
Supplement: Translation

# Translation: Visceral Obesity and High Systolic Blood Pressure as the Substrate of Endothelial Dysfunction in Obese Adolescents

**Authors:** Maria Fernanda Hussid, Felipe Xerez Cepeda, Camila P. Jordão, Rafaela R. P. Lopes-Vicente, Leslie Virmondes, Keyla Y. Katayama, Ezequiel F. de Oliveira, Luis V. F. Oliveira, Fernanda Marciano Consolim-Colombo, Ivani Credidio Trombetta

**Affiliations:** 1 São PauloSP Brazil Universidade Nove de Julho (UNINOVE), São Paulo, SP - Brazil.; 2 São PauloSP Brazil Instituto do Coração (InCor), Hospital das Clínicas da Faculdade de Medicina da Universidade de São Paulo, São Paulo, SP - Brazil.; 3 AnápolisGO Brazil Centro Universitário de Anápolis (UniEvangélica), Anápolis, GO - Brazil.

**Keywords:** Adolescent, Obesity, Metabolic Syndrome, Hypertension, Diabetes, Waist Circumference, Sleep Apnea Obstructive, Endothelium, Risk Factors

## Abstract

**Background:**

Obesity affects adolescence and may lead to metabolic syndrome (MetS) and endothelial dysfunction, an early marker of cardiovascular risk. Albeit obesity is strongly associated with obstructive sleep apnea (OSA), it is not clear the role of OSA in endothelial function in adolescents with obesity.

**Objective:**

To investigate whether obesity during adolescence leads to MetS and/or OSA; and causes endothelial dysfunction. In addition, we studied the possible association of MetS risk factors and apnea hypopnea index (AHI) with endothelial dysfunction.

**Methods:**

We studied 20 sedentary obese adolescents (OA; 14.2±1.6 years, 100.9±20.3kg), and 10 normal-weight adolescents (NWA, 15.2±1.2 years, 54.4±5.3kg) paired for sex. We assessed MetS risk factors (International Diabetes Federation criteria), vascular function (Flow-Mediated Dilation, FMD), functional capacity (VO^2^peak) and the presence of OSA (AHI>1event/h, by polysomnography). We considered statistically significant a P<0.05.

**Results:**

OA presented *higher waist* (WC), body fat, triglycerides, systolic (SBP) and diastolic blood pressure (DBP), LDL-c and lower HDL-c and VO^2^peak than NWA. MetS was presented in the 35% of OA, whereas OSA was present in 86.6% of OA and 50% of EA. There was no difference between groups in the AHI. The OA had lower FMD than NWA (6.17±2.72 vs. 9.37±2.20%, p=0.005). There was an association between FMD and WC (R=-0.506, p=0.008) and FMD and SBP (R=-0.493, p=0.006).

**Conclusion:**

In adolescents, obesity was associates with MetS and caused endothelial dysfunction. Increased WC and SBP could be involved in this alteration. OSA was observed in most adolescents, regardless of obesity. (Arq Bras Cardiol. 2021; 116(4):795-803)

## Introduction

Obesity has been increasing rapidly worldwide and is considered a risk factor for chronic noncommunicable diseases. Children and adolescents have been seriously impacted by this trend, particularly in developing countries, according to the World Health Organization (WHO).^1^ When assessing the nutritional status of school children aged 13 to 17 years, using the body mass index (BMI) for age, it was found that 23.7% of the male population is overweight, and 8.3% are obese.^2^ The concern about the increased prevalence of obesity in the children and adolescents lies in the fact that it may be a predictor of adult obesity, leading to increased risk of chronic diseases such as type 2 diabetes, metabolic syndrome (MetS) and cardiovascular diseases (CVD).^3^

Indeed, it is clear in the literature that obesity is positively associated with incident MetS.^4^ Studies carried in pubertal adolescents have demonstrated a prevalence of MetS ranging from 25 to 30%.^5^ In this study they found that waist circumference (WC) was a predictor of MetS, and an 11% increase in the risk of MetS was detected for each 1 cm added to abdominal circumference.^5^

It is well established that the earliest marker of atherosclerosis is endothelial dysfunction,^6^ which may be found in both hypertension and atherosclerosis. Endothelial dysfunction is also involved in physiological and pathological processes, including inflammation, insulin resistance and obesity, among other disorders.^6^

Flow-mediated dilation (FMD) by ultrasonography is a widely used noninvasive method to assess endothelium function, which may be a predictor of cardiovascular events in both asymptomatic and established cardiovascular disease (CVD) subjects. A change in FMD may be of prognostic value in humans.^7^

Respiratory sleep disorders are among the many consequences of obesity, including obstructive sleep apnea syndrome (OSA). OSA is the most common sleep-disordered breathing syndrome, with a prevalence of 1 to 4% in childhood, which peaks at the 2 - 8year-old age group.^8^ In children with obesity this percentage may reach 36%.^9^ OSA has been correlated with obesity, providing a mild and chronic inflammatory environment. Patients with OSA experience hypoxic episodes and recurrent arousals during sleep due to increased sympathetic nervous system activity.^9^ Trombetta et al.^10^ have found that MetS and OSA patients had higher blood pressure (BP) levels than those with MetS without OSA. Increased sympathetic activity and impairment of baroreflex control were observed in these patients with OSA associated with MetS.^10^ The association of obesity with OSA could increase the risk of endothelial dysfunction.^11^

In the present study, obese adolescents were compared with normal-weight adolescents regarding anthropometry, body composition, biochemical parameters, vascular reactivity, and sleep apnea. Our purpose was to investigate whether obesity during adolescence: 1) leads to MetS and/or OSA; and 2) causes endothelial dysfunction. In addition, we studied the possible association of MetS risk factors or apnea hypopnea index (AHI) with endothelial dysfunction.

## Methods

### Ethics Committee

The study was approved by the Research Ethics Committee of the Nove de Julho University (UNINOVE) under number 973.013, CAAE: 41899215.0.0000.5511. The adolescents’ parents or guardians were informed about the study procedures performed and gave written informed consent. The adolescents were also informed about all the procedures performed and provided written assent.

### Subjects

This was a cross-sectional study. Adolescents aged between 12 to 17 years, attending the Adolescent Outpatient Clinic of Universidade Nove de Julho (UNINOVE), were invited to participate in the study according to inclusion/exclusion criteria. Were included in the study post-pubertal adolescents according to Tanner staging (M4 girls or menarche and G4 boys),^12^^,^^13^ normal-weight or obese according to WHO BMI classification (over two standard deviations for obesity) for boys and girls, physically inactive, and not under dietary or drug treatment for obesity, with or without MetS. Exclusion criteria were adolescents who were not at the post-pubertal stage, overweight, and those overweight and with suspected or confirmed genetic syndromes or neuroendocrinological disorders such as uncontrolled hypothyroidism and type 1 diabetes. Patients with eating disorder (anorexia nervosa, bulimia nervosa, or unspecified eating disorder) were also excluded. A total of 20 obese adolescents (OA) and 10 normal-weight adolescents (NWA) were studied.

The International Diabetes Federation (IDF) criteria were used for diagnosis of MetS. Central obesity was defined as WC ≥94cm for men and ≥80 cm for women, plus two of these four diagnostic criteria: (1) high-density lipoprotein cholesterol (HDL-c) <40 mg/dL (<1.03 mmol/L) in men and <50 mg/dL (<1.29 mmol/L) in women; (2) fasting glucose level ≥l00 mg/dL (≥5.6 mmol/L); (3) fasting triglyceride level (TG) ≥150 mg/dL (>1.69 mmol/L); and (4) systolic blood pressure (SBP) ≥130 mmHg and diastolic blood pressure (DBP) ≥ 85 mmHg.^14^^,^^15^

### Measures

#### Anthropometric Measurements and Body Composition

Weight and height were assessed, and body mass index (BMI) was calculated. BMI was expressed as standard deviation scores (Z-score); normal weight was defined as a z-score between −2 and +1; overweight was defined as a z-score between +1 and +2; and obesity >+2. Assessment of body composition was performed by bioelectrical impedance analysis (RJL, Quantum II model, Clinton Twp, Mi, USA). WC and neck circumference (NC) were measured as previously described.^16^^,^^17^

#### Blood Pressure

SBP and DBP were measured with appropriate cuff size.^18^^–^^20^

#### Serum Analysis

Blood samples were collected after a 12-hour overnight fast. Concentrations of glucose, TG, total cholesterol, HDL-c, low density lipoprotein-cholesterol (LDL-c), TG/HDL-c ratio and LDL-c/HDL-c ratio were determined.

### Nocturnal Polysomnography

A whole-night polysomnography (standard monitoring - level 1) was performed using an ambulatory sleep analysis system (Embla Somnologica Studio - EMBLA A10, version 3.1.2.; Flagahf Medical Devices, Iceland), as previously described.^21^^,^^22^ As there were 12-year-old adolescents in the study, we used the American Academy of Sleep Medicine (AASM) criteria for OSA classification for children.^23^ The OSA was defined as an AHI >1 event/hour; an AHI ≥1-4.99 was considered mild OSA; an AHI of 5-9.99 was considered moderate OSA; and AHI ≥10 severe OSA.^9^

In children an apnea is scored when peak signal excursions drop by ≥ 90% of pre-event baseline. Hypoventilation is scored when the arterial CO^2^ (or surrogate) is > 50 mm Hg for > 25% of total sleep time. The AHI was calculated as the total number of respiratory events (apneas plus hypopneas) per hour of sleep. The arousal index was defined as the average number of arousals per hour of sleep. Oxygen desaturation (SaO^2^ nadir) was defined as the lowest hemoglobin oxygen saturation recorded by pulse oximetry.^23^

### Cardiopulmonary Exercise Testing (CPET)

CPET was performed on a treadmill, connected to a system composed of a gas analysis module, coupled to a flow module / wave analyzer in a breath-by-breath mode (BreezeCardiO2 System microcomputer; Medical Graphics Corporation-MGC, St. Paul, Mo, USA) and using a ramp protocol. The CPET allow to measure the functional capacity (VO^2^peak) as maximum VO^2^ attained at the end of the test.^24^^,^^25^

### Reactive Hyperemia

#### Flow-Mediated Dilation (FMD)

FMD was performed with high-resolution vascular ultrasound (Vivid i, GE Medical Systems, Tirat Carmel, Israel), by measuring the vessel dilation (endothelium-dependent dilation) of the brachial artery, as previously described.^26^ Briefly, subjects lay at rest for at least 10 minutes and a first resting scan was recorded. Then, an increased flow was induced by inflation of a sphygmomanometer cuff, located distally to the brachial artery in the forearm, to supra-systolic pressure (about 20 to 30 mmHg) for 5 minutes. The cuff was emptied and the flow and dilatation of the vessel, provided by the shear stress, was recorded. The difference between the basal diameter and the diameter after dilation was evaluated.

#### Reactive Hyperemia Index (RHI) by Peripheral Arterial Tonometry

Endothelial function was assessed by measuring the RHI by peripheral arterial tonometry (Endo-PAT2000; Itamar Medical, Caesarea, Israel) as previously described.^27^ This method evaluates microvascular endothelial function.^28^

For assessment of both FMD and RHI, adolescents were instructed to fast for 4 to 6 hours, and to refrain from caffeine, chocolate, fatty foods, and exercise on the day of the examination.

### Statistical Analysis

Statistical analysis was performed using the SPSS 20 Statistics program (IBM Corp., Armonk, NY, USA). The sample size was calculated using the website http://www.openepi.com. We took into account an 80% power, with a type 1 two-tailed error of 0.05. We used endothelial function variables (RHI and FMD) as the primary outcome. We chose the largest number of subjects, 30 adolescents for the study. Normality of the samples was tested by the Kolmogorov - Smirnov test. The parametric variables were expressed as mean ± standard deviation (SD) and nonparametric variables were expressed in median and interquartile range. The categorical data were described in absolute value and percentage of the total sample. The parametric variables of the OA and NWA groups were compared by independent Student's t-test while the non-parametric variables were compared by the Mann-Whitney test. Categorical variables were analyzed using the chi-square test and Pearson's correlation was used to analyze the correlation between variables of risk factors such as WC and BP and percentage of FMD. Probability values of *P*<0.05 were considered statistically significant.

## Results

We initially recruited 56 adolescents; 26 of them were excluded – nine of them were Tanner scale I, II or III; five were overweight; five had endocrine disorders; one used medication and six declined to participate in the study. Our final sample was composed by 30 adolescents. Thus, we studied 20 OA (10 male) and 10 NWA (5 male) (Figure 1). Seven adolescents of the OA group had MetS (35%) and no NWA had MetS (Figure 2).

**Figure 1:** Flowchart of patient recruitment; OA: obese adolescents; NWA normal-weight adolescents.

**Figure 2:** The percentage of obstructive sleep apnea (OSA) and metabolic syndrome (MetS) in adolescents with obesity (OA) and normal-weight (NWA).

In Table 1, we describe the anthropometric and body composition measurements. Both groups were similar in sex distribution and height. As expected, OA had higher weight, BMI, NC and WC. Regarding body composition, there was a lower percentage of body water and lean body mass and higher fat body mass in OA.

**Table 1:** Anthropometric and body composition measurements of obese and normal-weight adolescents

	OA (n=20)	NWA (n=10)	p
Sex (M/F)	10/10	5/5	1
Age (yrs)	14.2±1.6	15.2±1.2	0.075
Weight (kg)	100.1±20.3	54.4±5.3	<0.001
Height (m)	1.67±0.08	1.65±0.6	0.760
BMI (kg/m²)	35.9±6.2	19.9±1.8	<0.001
NC (cm)	38.3±3.6	31.9±1.8	<0.001
WC (cm)	107.9 [100-114.5]	67.5 [66.4-73.7]	<0.001
Body water (%)	45.4±3.8	56.5±4.6	<0.001
Fat body mass (%)	38±5.2	22.9±6.3	<0.001
Lean body mass (%)	62±5.2	77.1±6.3	<0.001

Parametric data presented as mean ± standard deviation. Non-parametric data presented as median and interquartile range. OA: obese adolescents; NWA: normal-weight adolescents; BMI: body mass index; NC: neck circumference; WC: waist circumference.

Data on cardiovascular risk factors in the OA and NWA groups are shown in Table 2. There were no differences in HDL-c or glycaemia between the groups. Compared with NWA, OA had higher SBP and DBP levels, as well as higher levels of TG, LDL-c, TG/HDL-c ratio, non-HDL, LDL/HDL-c ratio, and total cholesterol. In CPET, the OA group showed lower VO^2^peak compared to NWA. Results of polysomnography revealed that lower minimum O^2^ in OA compared with NWA. No differences in arousal index and AHI were found between groups (Table 2). However, most OA (86.6%) and 50% of NWA had AHI ≥ 1 event/h (Figure 2).

**Table 2:** Cardiovascular risk factors in obese and normal-weight adolescents

	OA (n=20)	NWA (n=10)	p
SBP (mmHg)	120 [110-127.5]	110 [100-110]	0.001
DBP (mmHg)	75 [70-80]	65 [60-70]	0.005
Glycaemia (mg/mL)	84.9±5.4	89.3 ±7.2	0.140
TG (mg/dL)	120.5±48.3	71.1±28.8	0.020
HDL-c (mg/dL)	41.2±7.7	48.4 ±10.7	0.079
TG/HDL-c ratio	3.1±1.6	1.6±1	0.011
LDL-c (mg/dL)	97.5±25.7	69.9±22.2	0.015
nHDL-c (mg/dL)	121.5±27.5	83.2±26.2	0.004
LDL/HDL-c radio	2.4±0.8	2.6±0.7	0.007
Total cholesterol (mg/dL)	162.7±28.7	132.5±24.1	0.016
**Nocturnal polysomnography**			
AHI (events/h)	5.6±3.8	3.1±3.4	0.121
Minimum O^2^ Sat (%)	90 [81-90]	92.5 [88.5-93]	0.026
Arousal index	50.6±18.1	50±9.3	0.943
**Cardiopulmonary exercise testing**			
VO^2^peak (mL/kg/min)	30.6±7.7	23.4±5.9	0.022

Parametric data expressed as mean ± SD. Non-parametric data expressed as median and interquartile range. OA: obese adolescents; NWA: normal-weight adolescents; SBP: systolic blood pressure; DBP: diastolic blood pressure; TG: triglycerides; HDL: high-density lipoprotein; LDL: low-density lipoprotein.

In Figure 3, we present the prevalence of MetS risk factors according to the IDF.^15^ In Figure 4 - panel A, we showed the FMD analyses. In one participant of the OA group, bifurcation of the brachial artery was detected, and we decided to exclude this record from the analysis. The analyses of the FMD showed that OA had lower vascular reactivity of large arteries compared to NWA (6.17±2.72% vs. 9.37±2.20%, p=0.005). Given this, we further explored the association between FMD with the risk factors for MetS, and found an associated between WC and FMD (R=-0.506, p=0.008; Figure 4 - panel B), and between SBP and FMD (R=-0.493, p=0.006; Figure 4 - panel C).

**Figure 3:** Percentage of risk factors for metabolic syndrome (MetS) in adolescent with obesity; WC: waist circumference; TG: triglycerides; HDL: high-density lipoprotein; SBP: systolic blood pressure; DBP: diastolic blood pressure.

**Figure 4:** Reactive hyperemia by flow-mediated dilation (FMD) (Panel A); correlation coefficient between FMD and waist circumference (WC) (Panel B); correlation coefficient between FMD and systolic blood pressure (SBP) in obese adolescents (OA) and normal-weight adolescents (NWA) (Panel C). * p<0.005.

## Discussion

The main finding of the present study is that OA have endothelial dysfunction, indicated by a decreased vascular reactivity. Moreover, based on correlation analyses, we can suggest that the WC and the SBP levels can be predictors of this dysfunction.

Obesity and MetS increase the risk of endothelial dysfunction, and OSA contributes to this worsening. However, despite the high prevalence of OSA in OA, there were no differences in the presence of OSA and the AHI between the groups studied. We can suggest that in adolescents other factors besides obesity, such as allergic rhinitis, asthma, and adenotonsillar hypertrophy, may contribute to the OSA. These findings have been found in other studies.^29^ Therefore, in our study, impaired FMD was not associated with OSA, and the presence of OSA could not be attributed to obesity or MetS. We speculate that MetS potentiated endothelial dysfunction in the obese group, since 35% of obese patients in our sample had MetS.

Atherosclerosis and clinical manifestations of CVD have their origin in childhood,^30^ and their early detection is very important for its prevention. Endothelial dysfunction is considered an early sign of atherosclerosis in children with CVD risk factors, and can be reversed by interventions to decrease cardiovascular risk.^30^

FMD with reactive hyperemia is a non-invasive method that evaluates nitric oxide (NO)-mediated endothelium-dependent vasodilation and is a suitable diagnostic method for the age group studied.^31^ A meta-analysis identified that a 1% increase in FMD increases the future risk of cardiovascular events by 13%.^3^ There is evidence that obese children and adolescents have lower vascular compliance and distensibility than normal-weight counterparts.^3^ This could explain the higher blood pressure levels found in OA.

A study in adults reported a FMD of 9.4% ± 4.7%.^32^ A meta-analysis by Dias et al.^3^ identified a FMD of 6.0% ± 0.69% in adolescents with obesity, compared to 12.32% ± 3.14% in NWA.^3^ This data corroborates our findings, showing an impaired vascular reactivity in OA compared with NWA (6.17 ± 2.72% and 9.37 ± 2.20%, respectively).

FMD is an indirect measure of NO bioavailability,^26^^,^^28^^,^^32^ since it simulates an ischemic environment, and later, vasodilation. Vessel occlusion leads to the release of adenosine, endothelium-derived hyperpolarizing factor, hydrogen ions, among other substances, with the aim of restoring blood perfusion through microcirculation dilation. In this method, when the cuff is deflated, circulation is restored with increased blood supply to the ischemic region, causing “reactive hyperemia”. The shear stress caused by the increase in blood flow and its velocity leads to the release of vasodilatory substances by the endothelium, such as NO, via activation of the endothelial NO synthase (eNOS) enzyme, leading to vascular smooth muscle relaxation and consequent increase in arterial diameter. A lower relaxation capacity leads to endothelial dysfunction.^26^^,^^28^^,^^32^ A lower vasodilation may occur in boys than in girls and there is variation of endothelial function during the menstrual cycle.^28^ Thus, we performed the tests in the first stage of the menstrual period. EndoPAT ® was evaluated by Radke et al. in relation to pubertal staging.^33^ A lower RHI was observed in the prepuberty in comparison with those at Tanner IV-V puberty stages, with an index ranging from 1.11 to 1.70. The cut off for adults is 1.35, which could be used to identify those individuals with endothelial dysfunction in the microcirculation. This technique was developed to be examiner-independent. It is known that, due to location of the cuff to be inflated, the vasodilation of microcirculation obtained is not totally dependent on NO. Therefore, while EndoPAT® measures the endothelial function of the microcirculation, the FMD evaluates the endothelial function of the conductance arteries. It is possible that the results are comparatively discrepant, but complementary, since these methods evaluate different systems.^28^ In the adolescents evaluated in this study, there was no difference in this measure between OA and NWA, so we could not identify complementarity between EndoPAT® and FMD.

Another relevant factor in the study was the correlation between WC and vascular reactivity. An increased WC is a risk predictor for CVD,^15^^,^^34^ and this is named as “visceral adiposity syndrome”.^4^^,^^34^ With the increase of the visceral adiposity, there is an increase in pathogenic fat depots and worsening of the vascular reactivity. Visceral fat distribution is a predictive factor for hypertension, greater than the generalized fat increase itself. Sympathetic nervous system seems to be related to different components of visceral adiposity syndrome, generating a real increase in sympathetic activity^34^ and an increased risk of hypertension in these patients.

Although there was no difference in AHI between the two groups studied, there was a prevalence of 86.6% and 50% of OSA, and of 35% and 0% of MetS in OA and NWA, respectively. The greater presence of OSA and MetS may have contributed to the increase of SBP in this group, which may be modulated by increased sympathetic tonus. This has already been observed by Trombetta et al., who reported higher sympathetic activity and reduced baroreflex control in adult patients with MetS associated with OSA.^10^

In the current study, we observed that OA exhibited decreased VO^2^peak suggesting increased cardiovascular risk. Indeed, there is strong evidence that obesity is associated with a worse prognosis in adolescents with reduced functional capacity and presence of cardiometabolic comorbidities. Preventive measures are necessary in these individuals with endothelial dysfunction, stimulating physical activity and a healthy diet aiming to reduce WC and BP.

### Limitations

Our study has several limitations. First, considering that in girls there is variation of endothelial function during the menstrual cycle,^28^ and although we performed the test in the first stage of the menstrual cycle, some girls had just had menarche and, therefore, did not have menstrual regularity or knowledge of their cycle. Second, since there is no consensus on the criteria for diagnosing OSA at the age from 13 to 18 years, in the present study, like others,^35^^,^^36^ we assumed pediatric values. The criteria used up to 13 years were extended up to the age of 18, based on the AASM manual for Scoring of Sleep and Associated Events.^23^

## Conclusion

In the sample studied, obesity was an important risk factor for development of MetS and lead to endothelial dysfunction, which is the onset of atheroma plaque. In addition, increased WC and SBP are predictors of endothelial dysfunction in adolescents. OSA was detected in most adolescents, regardless of obesity.
